# Impact of workplace violence against psychological health among nurse staff from Yunnan-Myanmar Chinese border region: propensity score matching analysis

**DOI:** 10.1186/s12912-023-01402-w

**Published:** 2023-07-26

**Authors:** Changmian Ding, Lidan Li, Guizhi Li, Xuehua Li, Linli Xie, Zhizhou Duan

**Affiliations:** 1 The Medical Record Management Department, Dehong People’s Hospital, Yunnan, China; 2 The Nursing Department, Dehong People’s Hospital, Yunnan, China; 3 Preventive health service, Jiangxi provincial people’s Hospital, The First Affiliated Hospital of Nanchang Medical College, Nanchang, Jiangxi China

**Keywords:** Workplace violence, Psychological health, Nurse staff, Yunnan-Myanmar Chinese border region, Propensity score matching analysis

## Abstract

**Background:**

Owing to different social background factor in Yunnan-Myanmar Chinese border region, stressful working environment may lead to extra psychological burden among nurse staff in China. However, the prevalence of workplace violence and its effect on psychological characteristics among nurse staff are still unclear. This study aims to explore the effect of workplace violence against psychological health among nurse staff from Yunnan-Myanmar Chinese border region.

**Methods:**

A cross-sectional survey was conducted among 18 local governmental hospitals in Dehong districts. Participants were 1,774 nurses. Psychosocial characteristics were screened by sleep quality, the 9-item Patient Health Questionnaire for depressive symptoms, the generalized anxiety disorder-7 for anxiety symptoms, the Connor Davidson Resilience Scale – 10 item for resilience, the multidimensional scale of perceived social support for social support, the Chinese version of Work place Violence Scale for workplace violence. Propensity score matching and multivariate linear regression were applied to analyze the data.

**Results:**

The nurse staff with workplace violence have a higher risk of bad sleep quality (b = -0.883, 95%CI = [-1.171, -0.595]), anxiety symptoms (b = 2.531, 95%CI = [2.031, 3.031]) and depressive symptoms (b = 3.227, 95%CI = [2.635, 3.819]), loneliness (b = 0.683, 95%CI = [0.503, 0.863]), perceived cognitive deficits (b = 1.629, 95%CI = [1.131, 2.127]), poor resilience (b = -2.012, 95%CI = [-2.963, -1.061]), and poor social support (b = -5.659, 95%CI = [-7.307, -4.011]).

**Conclusions:**

Preventing workplace violence can improve mental health outcomes significantly among nurse staff, including loneliness, perceived cognitive deficits, anxiety symptoms, depressive symptoms, sleep quality, resilience and social support.

## Introduction

Workplace violence (WPV) is widely recognized as a significant issue in negative medical work environments worldwide [1], and nurse staff are often the primary victims. Nurse staff members are known to be at high risk of WPV [2]. A meta included 136 international studies, related to Asian, European, etc., present that 36.4% of nurse staff experienced physically assaulted, and 67.2% reported have been nonphysical assaults [3]. During the normalized COVID-19 epidemic prevention and control of China, nurse staff members have been particularly vulnerable to workplace violence due to heavy workloads and stressful work environments [4], which can significantly impact their psychosocial characteristics.

Workplace violence can cause a range of health problems among nurse staff members. Theoretical evidence suggests that exposure to violence can weaken personal stress regulation, leading to negative mental reactions such as sadness, anger, and fear [5, 6]. Previous studies have also found that nurse staff members who are exposed to workplace violence are more likely to experience symptoms of anxiety and depression [7]. A meta-analysis has shown that exposure to workplace violence is associated with various sleep problems [8, 9]. In China, several studies have reported that a significant number of medical staff members experience symptoms of anxiety, depression, and burnout, among other psychological problems [10–12]. Such psychological problems may have both short-term and long-term effects on the mental health of nurse staff members [13]. In light of these findings, it is crucial to assess and attend to the mental health needs of nurse staff members who have experienced workplace violence, providing timely and effective psychological assistance services.

Workplace violence can have varying prevalence and impact on the mental health of nurse staff members in different regions of China, particularly those with multi-ethnic and multi-neighboring settings. However, research in these areas remains limited, especially in Chinese border regions with diverse ethnic groups. Therefore, our study aims to contribute to the existing literature by investigating the effects of workplace violence on the mental health outcomes of nurse staff members in the Yunnan-Myanmar Chinese border region, where unique challenges related to healthcare worker safety and well-being may arise. In addition, previous studies have primarily used linear or logistic regression models to explore the associations between different variables. However, by comparison with randomized controlled trials (RCTs), these models may only control for confounding factors to a limited extent. In recent research, propensity score matching (PSM) analysis has been proposed as an alternative method to address such issues [14, 15]. PSM involves dividing samples into treatment and control groups and using propensity scores to match their baseline socio-demographic variables [16]. This approach ultimately excludes unmatched samples and includes matched samples for final analysis. While previous studies have utilized regression analyses to examine the relationship between workplace violence and mental health outcomes, few studies have employed propensity score matching as a method of analysis.

Previous studies have primarily focused on the impact of workplace violence on mental health outcomes such as PTSD, depression, anxiety, and burnout among healthcare workers. However, there is still limited research on the impact of workplace violence on other mental health outcomes, such as loneliness and perceived cognitive deficits, which are important indicators of psychological well-being. This study aims to address this gap by examining the impact of workplace violence on psychosomatic outcomes, including loneliness, perceived cognitive deficits, anxiety symptoms, depressive symptoms, sleep quality, resilience, and social support among nurse staffs from the Yunnan-Myanmar Chinese border region. To investigate the potential differences in the effects of workplace violence on mental health outcomes, this study employs a combination of Propensity Score Matching (PSM) and regression analysis. Unlike prior research that has relied solely on regression analysis, the PSM method is used to obtain matched data, which is subsequently analyzed using regression analysis to examine the relationships between variables. To summarize, this study aims to examine the impact of workplace violence on mental health outcomes among nurse staffs from the Yunnan-Myanmar Chinese border region, by applying the PSM method. Specifically, this study aims to compare the effectiveness of PSM methods in matching confounders and the differences in outcomes between pre-matching and post-matching methods.

## Methods

### Participants and procedure

A cross-sectional study was conducted in Dehong districts, Yunnan province from July 11 to July 26, 2022. Nurse staff comes from all 18 local governmental hospitals in Dehong districts, were recruited to participate in this study. Dehong is an ethnic border district located in the southwest of China, with a population of approximately 1.32 million people as of the end of 2021 [17]. It shares a 503.8-kilometer-long border with Myanmar on its north, west, and south sides [18]. The convenience sampling method was applied to recruit the participants, who completed our questionnaires using Wenjuanxing software, the largest online questionnaire software in China. Trained researchers fully explained the purpose of the study to each participant, and the questionnaire link was distributed by the nursing department of each governmental hospital. The survey was anonymous, and participants were asked to complete the questionnaire independently. They could contact the researchers if they had any questions about the survey and were informed of their right to withdraw at any time. Most participants took approximately 7 min to complete the questionnaire. Ultimately, a total of 1965 nurse staff members were invited to participate, and 1774 completed the survey, resulting in a response rate of 90.3%. The Ethics Committee of Dehong People’s Hospital in China approved the study (Ethics Approval Number: DYLL-KY032).

### Inclusion criteria and sample size

Participants met the following inclusion criteria and then have been included in this study: (1) work at the 18 local governmental hospitals; (2) able to understand the content of questionnaire; (3) agreed to participants and provided informed consent; (4) were not mental illness, (5) were not student nurse. The cross-sectional survey formula was applied to calculate the sampling as follows:

$$\text{N} = \frac{{Z_{1 - \partial /2}^2 \times pq}}{{{d^2}}}$$

Z_1−α/2_ is the statistical value for significance testing, where α = 0.05 and its value is 1.96. p is the prevalence rate of psychological health problems, where q = 1-p, and d is the allowable error, where d = 0.2p. According to previous studies, the prevalence rate of psychological health problems among nurses were ranging from 6.2 to 36.9% [19]. In this study, the minimum value of 6.2% was used for calculations, which translates to a minimum of 1706 subjects considering a non-response rate of 10%.

### Measures

#### Socio-demographic variables

Basic socio-demographic characteristics such as age, sex, ethnic, marital status, residence, education level, only children, monthly income, and work experiences were collected. In addition, we recorded their weight and height, and used these measurements to calculate their BMI score (BMI = Weight [kg] /Height^2^ [m]). The BMI group was classified into four groups: Underweight (< 18.5), Normal (18.5 ~ 24.9), Overweight (25.0 ~ 29.9), and Obese (> 30.0).

#### Workplace violence

Workplace violence were assessed by the Chinese version of Work place Violence Scale (WVS) [20], which consists of five-dimension items with physical assault (PA), emotional abuse (EA), threats (T), verbal sexual harassment (VSH), sexual abuse (SA) (e.g., In the past year, have you encountered the physical assault violence from patients or patients’ relatives? Including pushing, biting, beating, spitting). Each item was scored on a scale from 0 to 3 (0 = never, 1 = one time, 2 = two or three times, 3 = more than three times). The total score for the scale was calculated by summing each item and then dividing participants into two categories: those who had experienced workplace violence (score from 1 to 15) and those who had not (score of 0). This scale has been confirmed good reliability and validity in China [21]. The Cronbach’s α = 0.76 in this study.

#### Loneliness

Loneliness was assessed using the Three-Item Loneliness Scale [22], which is a 3-point Likert scale consisting of three items that ask participants to rate how often they feel they lack companionship. Response options include “hardly ever,“ “some of the time,“ and “often.“ The total score for the scale is calculated by summing each item, resulting in a score range of 3 to 9. Higher scores indicate a greater level of loneliness. The Cronbach’s α = 0.83 in this study.

#### Sleep quality

Sleep quality was assessed by the single-item sleep quality scale (SQS) [23]. Participants were asked to rate their overall sleep quality during the past seven days on an 11-point scale ranging from 0 (terrible) to 10 (excellent), with higher scores indicating better sleep quality. The SQS is a single-item questionnaire that has been found to have appropriate measurement characteristics for evaluating sleep quality, compared to longer questionnaires such as the Morning Questionnaire-Insomnia and Pittsburgh Sleep Quality Index (PSQI) [23]. The instrument has been reported to have a good reliability and validity, and it has been used in various published studies [24, 25], including research conducted in China [26, 27].

#### Perceived cognitive deficits

Perceived cognitive deficits were assessed using the Perceived Deficits Questionnaire (PDQ-5) [28]. This questionnaire consists of five questions related to attention, concentration and planning, organization, retrospective memory, and prospective memory. Participants were asked to rate their level of agreement with each item on a scale ranging from “never” (scored 0) to “always” (scored 4). The total score for the questionnaire is calculated by summing each item, with higher scores indicating more severe perceived cognitive deficits. The Cronbach’s α = 0.85 in this study.

#### Anxiety symptoms

The generalized anxiety disorder-7 (GAD-7) [29] was used to measure anxiety symptoms in this study. This scale consists of seven items that ask participants to rate how often they have experienced specific symptoms of anxiety in the past two weeks, on a 4-point scale ranging from “not at all” to “nearly every day.“ The total score for the scale ranges from 0 to 21, with higher scores indicating more severe anxiety symptoms. The Chinese version of GAD-7 have been confirmed good validity and reliability [30]. The Cronbach’s α = 0.93 in this study.

#### Depressive symptoms

Depressive symptoms were assessed using the 9-item Patient Health Questionnaire (PHQ-9) [31]. This scale consists of nine items that ask participants how often they have experienced specific symptoms of depression in the past two weeks, on a 4-point Likert scale ranging from “not at all” to “nearly every day.“ Scores for each item range from 0 to 3, depending on the response. The total score for the scale ranges from 0 to 27, with higher scores indicating more severe depressive symptoms. The Chinese version of PHQ-9 have shown good validity and reliability in China [32, 33]. And the Cronbach’s α = 0.91 in this study.

#### Resilience

Resilience was assessed using the Connor-Davidson Resilience Scale-10 item (CD-RISC-10) [34]. This scale consists of ten items that ask participants to rate the extent to which they agree with specific statements about their ability to cope with adversity, on a 5-point scale ranging from “never true” to “always true.“ Scores for each item range from 0 to 4, depending on the response. The total score for the scale is calculated by summing the scores for each item, with higher scores indicating stronger resilience levels. The CD-RISC-10 has been found to have good validity and reliability in China [35]. And the Cronbach’s α = 0.94 in this study.

#### Social support

Social support was assessed by the multidimensional scale of perceived social support (MSPSS) [36]. It is containing 12 items with response on seven-point Likert scale, ranged from 1 (strongly disagree) to 7 (strongly agree). The sum score were calculated by adding each item, with higher scores indicting higher level of social support. The Chinese version of this scale have been used in Chinese population [37]. And the Cronbach’s α = 0.96 in this study.

### Statistical analysis

#### Descriptive analysis

Qualitative data were presented as numbers and percentages (N/%), while quantitative data were presented as mean ± standard deviation (SD). The chi-square test and t-test were used to assess differences in basic sociodemographic variables between participants who experienced workplace violence and those who did not.

#### PSM analysis

In order to control confounding covariates, propensity score (PS) is a statistic that calculates the conditional probability of being assigned to a specific treatment, given a set of observed covariates [38], which can be used to decrease the impact of selection bias. We used PSM analysis to match baseline socio-demographic characteristics, including age, sex, ethnic, marital status, residence, education level, only children, monthly income and BMI. In this study, nurse with workplace violence as experiment group, and non-workplace violence nurse as control group, and Nearest neighborhood matching were used to matched baseline socio-demographic variables (caliper = 0.05), with a ratio of 1 (case) :1(control).

### Multivariate liner regression

Multivariate liner regression model was used to assess the effect of workplace violence on mental health outcomes before matching and after matching. All multivariate liner regression model also controlled basic socio-demographic covariates.

R version 3.6.2 were used to performed PSM analysis and other statistical analysis were conducted by SPSS version 22.0. P value were set at 0.05 in this study (two-tails). The Cronbach’s alpha coefficient (Cronbach’s α) was calculated to confirm the reliability of the questionnaires in this study. The Cronbach’s α ranges from 0 to 1, with higher values indicating greater internal consistency. Generally, a Cronbach’s α value of 0.70 or higher is considered acceptable for most research purposes [39, 40].

## Results

A total of 1,774 nurses were included in the statistical analysis, of whom 559 (31.5%) reported experiencing workplace violence in the past year. Table 1 presents the baseline sociodemographic characteristics and mental health outcomes of the participants. The majority of nurses were female (93.9%), of Han ethnicity (71.9%), married (67.6%), had a normal body mass index (64.0%), were only children (84.0%), lived in rural areas (60.4%), and had a monthly income of 3001–5000 RMB (44.1%). The average age of the nurses was 32.00 years old (SD = 7.99), and most had 5–9 years of nursing experience (33.4%). With regard to mental health outcomes, the mean scores for loneliness, sleep quality, perceived cognitive deficits, anxiety symptoms, depressive symptoms, resilience, and social support were 2.26 (SD = 1.55), 6.33 (SD = 2.44), 7.12 (SD = 4.27), 6.29 (SD = 4.32), 7.42 (SD = 5.13), 21.85 (SD = 8.28), and 62.60 (SD = 14.02), respectively.

**Table 1:** Socio-demographic characteristics and Mental health outcomes of participants (N = 1774)

Characteristic	Number	Percent (%)
Age (years)	32.00 ± 7.99	
20–24	185	10.4
25–29	674	38.0
30–34	452	25.5
35–39	171	9.6
40–59	292	16.5
Sex		
Women	1666	93.9
Men	108	6.1
Ethnic		
Han	1276	71.9
Others	498	28.1
BMI group		
Normal	1136	64.0
Thin	191	10.8
Overweight	341	19.2
Obese	106	6.0
Marital status		
Unmarried	517	29.1
Married	1200	67.6
Divorce/others	57	3.2
Residence		
Rural	1071	60.4
Urban	703	39.6
Education level		
High school or lower	614	34.6
Bachelor degree or above	1160	65.4
Only children		
Yes	283	16.0
No	1491	84.0
Income (monthly)		
3000 or lower	498	28.1
3001–5000	782	44.1
5001–7000	325	18.3
7000 or higher	169	9.5
Experience (years)	10.83 ± 8.55	
0–4	393	22.2
5–9	592	33.4
10–14	391	22.0
15–19	128	7.2
20–40	270	15.2
Mental health (Mean ± SD)		
Loneliness	2.26 ± 1.55	
Sleep quality	6.33 ± 2.44	
Perceived cognitive deficits	7.12 ± 4.27	
Anxiety symptoms	6.29 ± 4.32	
Depressive symptoms	7.42 ± 5.13	
Resilience	21.85 ± 8.28	
Social support	62.60 ± 14.02	

**Fig. 1:**
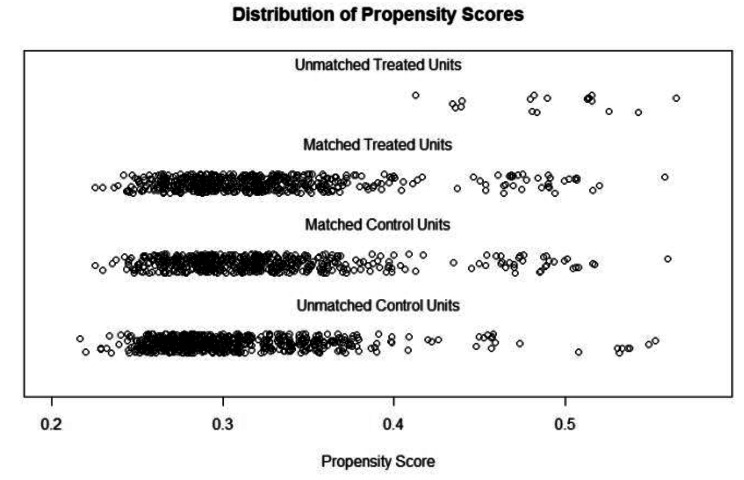
The distribution of propensity score before and after PSM analysis

**Fig. 2:**
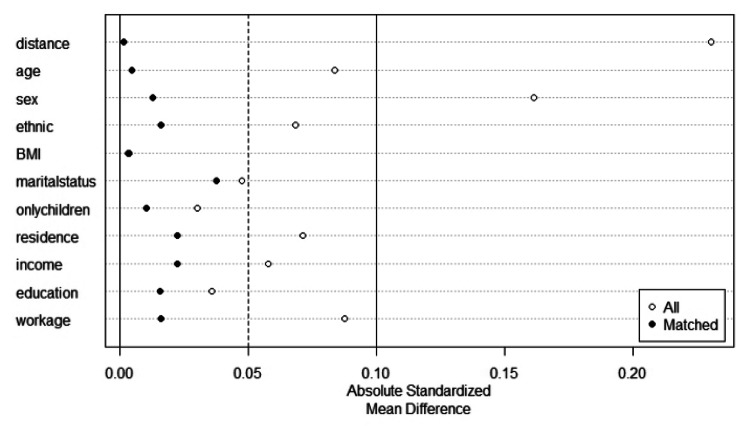
The absolute standardized mean difference before and after PSM analysis

In the propensity scores matching analysis, nurses who did not experience workplace violence were designated as the control group, while those who did were considered the treatment group. Figure 1 displays the distributions of propensity scores between unmatched samples and matched samples. In details, the total sample have been dropped from 1774 to 1082 (N non-workplace violence = 541, N workplace violence = 541), with a total of 692 unmatched cases excluded. The absolute standardized mean difference of basic socio-demographic characteristics is down from 0.230 to 0.002 before matching and after matching, indicting post-matched samples present a well covariate balance (See Figure 2). In addition, the result of Chi-square test and T test of basic socio-demographic characteristics separated by workplace violence before and after matching also have confirmed that the PSM analysis was performed well (Chi-square/T value and P value were decreased) (See Table 2).

**Table 2:** The socio-demographic characteristics of participants before and after PSM between workplace violence and non-workplace violence

Variables	Before PSM analysis (N = 1774)		χ^2^	*P*	After PSM analysis(N = 1082)		χ^2^	*P*
	Non-workplace violence (N = 1215)	workplace violence (N = 559)			Non-workplace violence (N = 541)	workplace violence (N = 541)		
Sex			14.75	< 0.001			0.06	0.805
Women	1159(69.6)	507(30.4)			505(49.9)	507(50.1)		
Men	56(51.9)	52(48.1)			36(51.4)	34(48.6)		
Ethnic			1.89	0.170			0.07	0.790
Han	886(69.4)	390(30.6)			383(50.3)	379(49.7)		
Others	329(66.1)	169(33.9)			158(49.4)	162(50.6)		
BMI group			0.70	0.873			0.07	0.995
Normal	776(68.3)	360(31.7)			350(49.9)	351(50.1)		
Thin	135(70.7)	56(29.3)			55(49.5)	56(50.5)		
Overweight	230(67.4)	111(32.6)			106(50.7)	103(49.3)		
Obese	74(69.8)	32(30.2)			30(49.2)	31(50.8)		
Marital status			2.02	0.364			1.66	0.436
Unmarried	365(70.6)	152(29.4)			144(49.7)	146(50.3)		
Married	809(67.4)	391(32.6)			373(49.6)	379(50.4)		
Divorce/others	41(71.9)	16(28.1)			24(60.0)	16(40.0)		
Residence			1.98	0.159			0.14	0.712
Rural	747(69.7)	324(30.3)			314(50.5)	308(49.5)		
Urban	468(66.6)	235(33.4)			227(49.3)	233(50.7)		
Education level			0.48	0.487			0.07	0.796
High school or lower	427(69.5)	187(30.5)			171(49.4)	180(50.6)		
Bachelor degree or above	788(67.9)	372(32.1)			365(50.3)	361(49.7)		
Only children			0.34	0.560			0.03	0.866
Yes	198(70.0)	85(30.0)			84(50.6)	82(49.4)		
No	1017(68.2)	474(31.8)			457(49.9)	459(50.1)		
Income (monthly)			6.46	0.091			3.04	0.385
3000 or lower	351(70.5)	147(29.5)			159(52.5)	144(47.5)		
3001–5000	540(69.1)	242(30.9)			224(49.0)	233(51.0)		
5001–7000	204(62.8)	121(37.2)			99(46.3)	115(53.7)		
7000 or higher	120(71.0)	49(29.0)			59(54.6)	49(45.4)		
	Mean ± SD	Mean ± SD	T	*P*	Mean ± SD	Mean ± SD	T	*P*
Age	31.78 ± 7.79	32.48 ± 8.40	-1.72	0.085	32.57 ± 7.94	32.60 ± 8.50	-0.08	0.938
Experience (years)	10.58 ± 8.35	11.37 ± 8.95	-1.80	0.072	11.37 ± 8.55	11.52 ± 9.04	-0.27	0.790

**Fig. 3:**
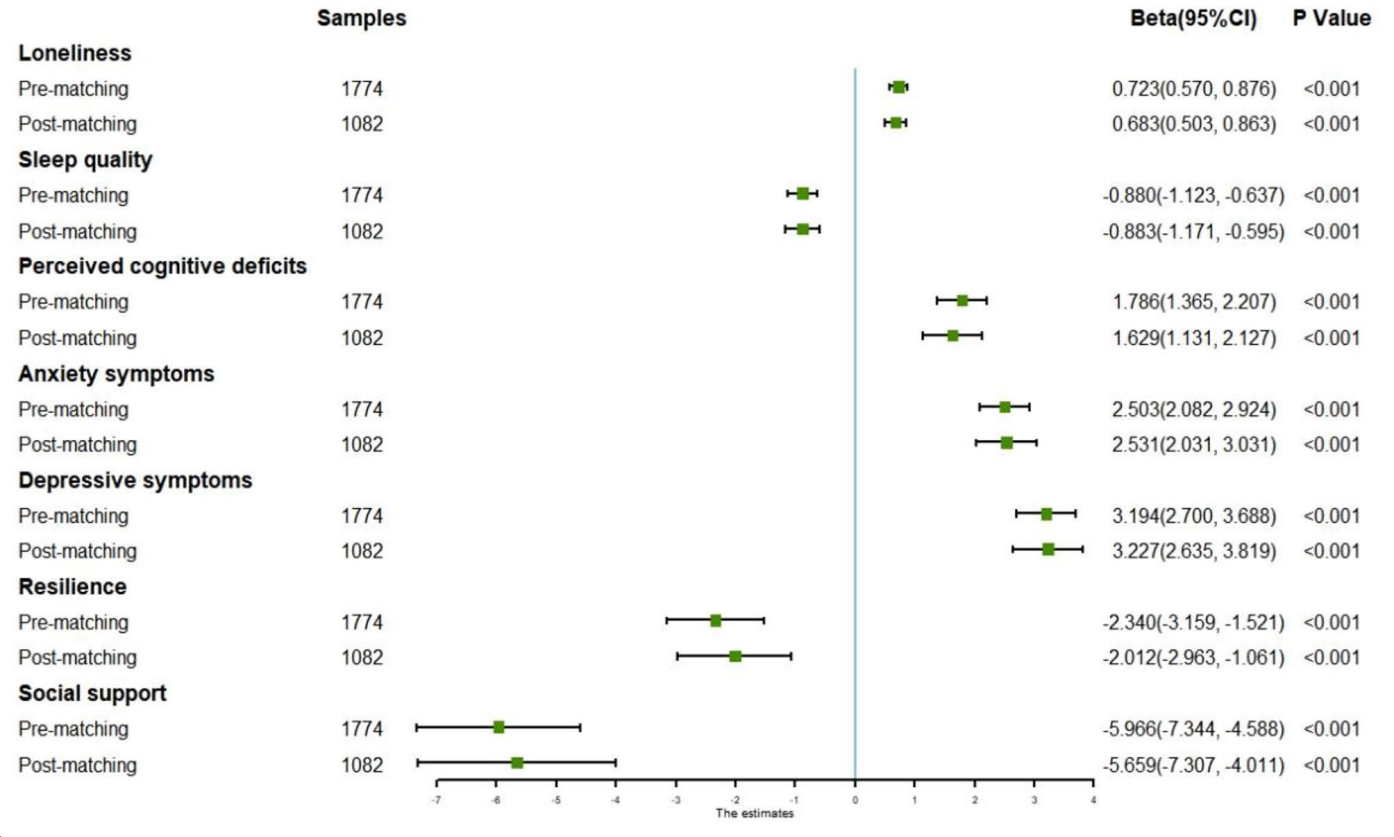
The effect of workplace violence on mental health outcomes before and after PSM analysis

Figure 3 exhibited the link between workplace violence and mental health outcomes with Pre-matching and Post-matching among nurse staff. In Pre-matching analysis, after controlling socio-demographic covariates, multivariate liner regression models revealed that nurse staffs with workplace violence have a higher risk of loneliness (b = 0.723, 95%CI = [0.570, 0.876]), poor perceived cognitive deficits (b = 1.786, 95%CI = [1.365, 2.207]), anxiety symptoms (b = 2.503, 95%CI = [2.082, 2.924]) and depressive symptoms (b = 3.194, 95%CI = [2.700, 3.688]). And workplace violence can negatively affect good sleep quality (b = -0.880, 95%CI = [-1.123, -0.637]), resilience (b = -2.340, 95%CI = [-3.159, -1.521]) and social support (b = -5.966, 95%CI = [-7.344, -4.588]). In Post-matching analysis, all covariates were matched well, and the result of multivariate liner regression show different with Pre-matching results. In details, the nurse staff with workplace violence was associated with sleep quality (b = -0.883, 95%CI = [-1.171, -0.595]), anxiety symptoms (b = 2.531, 95%CI = [2.031, 3.031]) and depressive symptoms (b = 3.227, 95%CI = [2.635, 3.819]), with higher beta value of liner regression models. Other mental health outcomes were also associated workplace violence significantly, with lower beta value of liner regression models including loneliness (b = 0.683, 95%CI = [0.503, 0.863]), perceived cognitive deficits (b = 1.629, 95%CI = [1.131, 2.127]), resilience (b = -2.012, 95%CI = [-2.963, -1.061]), and social support (b = -5.659, 95%CI = [-7.307, -4.011]).

## Discussion

In this study, we revealed that the adverse impact of workplace violence on psychosomatic outcomes and PSM analysis methods of control confounding factors show effectively. Results showed that workplace violence was significantly associated with higher levels of loneliness, sleep quality, perceived cognitive deficits, anxiety symptoms, depressive symptoms, reduced resilience, and decreased social support among nurses. Hospital administrators should take effective measures to prevent workplace violence and mitigate the mental stress associated with it, in order to avoid psychosomatic problems. In addition, this study is also the first to report the prevalence of workplace violence among nurse staff in the Yunnan-Myanmar Chinese border region. The rate of workplace violence was 31.5% in the past 12 months among nurse staff in this study. Several previous studies shown the different rate of workplace violence depending on the study design, timeframe, location, and other factors [41–43]. A meta-analysis shown that the rate of workplace violence was 62.4% for the whole of life among health work provider in China [44]. Lu’s study also revealed that 84.2% of frontline psychiatric nurses was experienced workplace violence [44]. However, the prevalence of workplace violence was 18.5% among health work provider [45]. Direct comparison cannot be done due to not locate any previous research in Yunnan-Myanmar Chinese border region. So, our findings highlight the need for targeted interventions to address workplace violence and its impact on the mental health of healthcare workers in the Yunnan-Myanmar Chinese border region.

Our results also revealed that workplace violence increased the likelihood of anxiety symptoms, depression symptoms, bad sleep quality, and it also decreased the level of resilience and social support among nurse staff, which consist of previous studies [46–49]. A cross-sectional study surveyed in China have shown that workplace violence plays a negative effect on nurse staff’s mental health and well-being [46]. A review contains 16 international researches revealed that nurse staff exposed to workplace violence can have a higher risk of poor quality of life welling-being, life satisfaction, depressive symptoms, occupational stress [47]. Furthermore, a survey carried out among healthcare professionals in China revealed that workplace violence could greatly diminish their perceived social support, ultimately resulting in mental health issues [50]. Similarly, another study conducted on Chinese nurses and general practitioners highlighted that experiencing workplace violence can decrease their resilience levels, ultimately causing symptoms of depression [51]. Conservation of resources theory have elaborated that the individual of resources are limited [52], and workplace violence can increase the discomfort of nurse staff, which further increased nurse staff’s resource consumption. And the new resources such as self-esteem, social support and resilience were hard to obtain for high exposure of nurse staff to workplace violence [48, 49]. Thus, poor mental health outcomes can be raised with the consumption of resource, and future study can examine more mental health outcomes and the relationship between workplace violence and them to provide related strategies to tackle workplace violence. Moreover, it is important to mention that the linear regression analysis conducted post-matching demonstrated a higher effect coefficient for workplace violence on anxiety and depressive symptoms compared to the pre-matching analysis, while the effect coefficient for workplace violence on resilience and social support was lower. This difference in regression coefficients may be attributed to the confounding factors such as age and sex that were controlled in the Propensity Score Matching (PSM) analysis. The accurate linear coefficients were displayed, especially when the actual impact of workplace violence on resilience, social support, anxiety, and depressive symptoms with covariates were perfectly balanced.

Consistent with previous research findings [8, 53, 54], our study found a positive association between workplace violence and poor sleep quality. A review of 119,361 participants across 15 countries demonstrated that experiencing physical, verbal, or sexual violence in the workplace was a predictor of sleep problems [8]. Similarly, a cross-sectional study of 550 nurses and nursing assistants revealed a significant association between workplace violence and headaches and poor sleep quality. Those exposed to physical abuse at work had over twice the risk of developing headaches and poor sleep quality [53]. Psychological stress after experiencing workplace violence may be a contributing factor leading to sleep disturbance and health issues [55]. Moreover, gender of the nurse staff was found to be related to impaired sleep quality [56]. However, gender was considered a covariate in this study to balance the relationship between workplace violence and sleep quality after post-matching. Consequently, the significant linear regression coefficient in pre-matching and post-matching indicated that the effect of workplace violence on sleep problems was underestimated.

Our findings on the relationship between workplace violence and loneliness also worth a mention. The results revealed that nurse staff with workplace violence were found to report to higher level loneliness. In line with prior findings, the general population have shown a strong association between loneliness and bullying or abuse [57, 58]. In the stage of normalized COVID-19 pandemic prevention and control, nurse staff exposed to negative interpersonal events can induce the sense of social alienation, and lead to the avoidance of social situation [59]. If the social situation were not altered, and this tough social situation could cause the feeling of loneliness among nurse staff. Previous studies have reported varying levels of loneliness among nurses of different genders[60]. Therefore, in this study, the age was controlled as a covariate to balance the relation between workplace violence and loneliness after post-matching. The significant linear regression coefficient in pre-matching and post-matching indicated that the effect of workplace violence on loneliness was overestimated.

In agreement with previous studies, poor perceived cognitive deficits was associated with workplace violence in this study. In shobhit’s research surveyed in India, the violent older adult tends to be of lower cognitive ability than non-violent older adults [61]. Similarly, Priscilla’s study revealed that the children with intimate partner violence have a lower cognitive ability score within a year, while it is not significantly within 10 years [62]. Lower cognitive ability are unfavorable factors of satisfaction nursing and safety nursing among nurse staff, which may further increase the possibility of workplace violence. To the best understating of present studies, the mechanism of cognitive ability and violence are still unclear. However, Priscilla and his colleges assume that the violence events can affect chronic biological stress, and in turn response to subsequent cognitive development. Future studies maybe test and verify it by experimental program. Furthermore, the inconsistent results obtained before and after matching indicated that the propensity score matching method is more robust in testing the effects of WPV compared to the simple regression method.

Consistent with aims, our findings also proved that PSM analysis is an important instrument to control covariate characteristics. By performed PSM analysis, the baseline socio-demographic characteristics were more comparable between workplace violence and non-workplace violence nurse staff. And it further leading to the distinguish coefficient of pre-matching and post-matching on multiple liner regression model, with higher/lower beta value of liner regression models. To achieve effectiveness, PSM analysis may be a functional approach to meet the exacting statistical requirements in further research.

Workplace violence has adverse effects on the well-being of nurses, highlighting the importance of taking steps to prevent it. The healthcare system in China has implemented a “safe hospital” policy that utilizes social media to promote positive images of nurses and raise awareness of their contributions [63]. Hospitals have also collaborated with public security departments to establish warning and defense systems to address workplace violence. Such strategies hold promises for effectively reducing instances of workplace violence against healthcare workers, including nurses [63, 64]. Additionally, our study suggests that hospitals should further take measures to enhance their aftermath management for nurses who have experienced workplace violence. Effectively strategies aim to improving work environment and accessibility of psychological counseling may be beneficial for decreasing the frequency of workplace violence and improving these nurse staffs’ psychological health. For example, nursing managers can conduct comprehensive measures to improve staff-patient relationship, such as creating a good nurse atmosphere, strength the accessibility of psychological counseling.

This study has some limitations that should be acknowledged. Firstly, as the data was collected using a cross-sectional design, it is difficult to establish causal relationships between variables. Future longitudinal studies should be conducted to further verify the validity of the findings. Secondly, although we employed PSM analysis to control for potentially confounding demographic variables, other confounding factors (such as nursing department and night shift schedules) may still exist. Future studies could build upon these findings by conducting a more in-depth analysis that includes these additional factors. Thirdly, our sample only includes participants from Dehong districts in Yunnan province, and as such, the representativeness of the results may not extend to nursing staff throughout China. Finally, participant information was derived from self-reporting, which may have introduced self-reporting bias due to participants concealing certain information.

## Conclusion

This study offers novel insights into the prevalence of workplace violence (31.5%) among nurse staff from Yunnan-Myanmar Chinese border region. Our findings also highlight the effectiveness of Propensity Score Matching as a statistical tool for controlling the impact of confounding factors and achieving more precise results. As such, future research could benefit from utilizing this approach to enhance precision even further. Furthermore, the data we have gathered presents a strong association between nurse staff exposed to workplace violence and experiences of loneliness, perceived cognitive deficits, anxiety symptoms, depressive symptoms, poor sleep quality, poor resilience, and lack of social support. These results emphasize the urgent need to implement preventative measures to address this pressing issue in the workplace.

## Data Availability

The datasets generated during and/or analysed during the current study are available from the corresponding author on reasonable request.`.

## Abbreviations

WPV: Workplace violence
RCTs: Randomized controlled trials
PSM: propensity score matching analysis
GAD-7: The generalized anxiety disorder-7
CDRISC-10: The Connor Davidson Resilience Scale – 10 item
PHQ-9: the 9-item Patient Health Questionnaire
